# Novel Crosslinked Sulfonated PVA/PEO Doped with Phosphated Titanium Oxide Nanotubes as Effective Green Cation Exchange Membrane for Direct Borohydride Fuel Cells

**DOI:** 10.3390/polym13132050

**Published:** 2021-06-23

**Authors:** Marwa H. Gouda, Noha A. Elessawy, Arafat Toghan

**Affiliations:** 1 Polymer Materials Research Department, Advanced Technology and New Materials Research Institute (ATNMRI), City of Scientific Research and Technological Applications City (SRTA-City), Alexandria 21934, Egypt; marwagouda777@yahoo.com; 2 Advanced Technology and New Materials Research Institute (ATNMRI), City of Scientific Research and Technological Applications City (SRTA-City), Alexandria 21934, Egypt; 3 Chemistry Department, Faculty of Science, South Valley University, Qena 83523, Egypt; arafat.toghan@yahoo.com; 4 Chemistry Department, College of Science, Imam Mohammad Ibn Saud Islamic University (IMSIU), Riyadh 11623, Saudi Arabia

**Keywords:** proton exchange membrane, polyvinyl alcohol, polyethylene oxide, direct borohydride fuel cell

## Abstract

A direct borohydride fuel cell (DBFC) is a type of low temperature fuel cell which requires efficient and low cost proton exchange membranes in order to commercialize it. Herein, a binary polymer blend was formulated from inexpensive and ecofriendly polymers, namely polyethylene oxide (PEO) and poly vinyl alcohol (PVA). Phosphated titanium oxide nanotube (PO_4_TiO_2_) was synthesized from a simple impregnation–calcination method and later embedded for the first time as a doping agent into this polymeric matrix with a percentage of 1–3 wt%. The membranes’ physicochemical properties such as oxidative stability and tensile strength were enhanced with increasing doping addition, while the borohydride permeability, water uptake, and swelling ratio of the membranes decreased with increasing PO_4_TiO_2_ weight percentage. However, the ionic conductivity and power density increased to 28 mS cm^−1^ and 72 mWcm^−2^ respectively for the membrane with 3 wt% of PO_4_TiO_2_ which achieved approximately 99% oxidative stability and 40.3 MPa tensile strength, better than Nafion117 (92% RW and 25 MPa). The fabricated membrane with the optimum properties (PVA/PEO/PO_4_TiO_2_-3) achieved higher selectivity than Nafion117 and could be efficient as a proton exchange membrane in the development of green and low cost DBFCs.

## 1. Introduction

A direct borohydride fuel cell DBFC is an electrochemical device for energy conversion, that uses nonexplosive and nontoxic reactants, provides high energy density, and can operate at low temperatures while empowering its application in portable sectors and transportation [1,2,3,4]. DBFCs provide electricity by liquid or gaseous oxidant reduction and borohydride ion (BH4−) oxidation whereas, sodium borohydride (NaBH4) is used as a nonhydrocarbon liquid fuel, thus avoiding carbon dioxide emission, as occurs in fuel cells fed by alcohol. Liquid hydrogen peroxide (H_2_O_2_) is favored over oxygen as an oxidant because it has quicker reduction kinetics and thus provides a higher power density, which broadens DBFC applications in oxygen free environments such as space and underwater environments [5,6,7].

A membrane is used as a separator in the fuel cell between the cathodic and anodic compartments which meanwhile allows ions transport in order to keep the charges balanced in the fuel cell. An anion-exchange membrane (AEM) can transfer OH− easily from the cathode to anode, but due to borohydride crossover, the fuel cell efficiency decreases. The cation exchange membrane (CEM) can reduce the borohydride crossover as a result of electrostatic repulsion occurring between the BH4− negative charges and the negative charges of the CEM backbone [5]; moreover, CEM allows the transportation of Na+ ions from the anode to the cathode. The Nafion family is the most perfluorinated CEM used in DBFCs [7,8,9] because it provides good mechanical and chemical stability and ionic conductivity [5,7,8]. However, Nafion membrane fabrication is expensive and requires a complex process, which limits its commercialization [10,11], meaning that its replacement by green and cost-effective polymeric membranes is essential [5,7,12]. However, membrane fuel cell development includes polymer sulfonation or polymer blending, and/or doping agent incorporation in the polymeric matrix, such as functionalized carbon materials and porous and functionalized inorganic materials to replace Nafion membranes [11,13]. Non-perfluorinated polymers, such as polyether ether ketone (PEEK), polystyrene (PS), polyarylene ether sulfone (PSU) and polybenzimidazole (PBI), are the most common polymers used to synthesize novel alternative polymeric membranes [11,12,13,14]. The synthesis of these nondegradable polymers requires toxic organic solvents, time, and temperature, thus making membrane synthesis costly, complex, and not ecofriendly. From an economic and technological point of view, using biodegradable, inexpensive, and green polymers, such as polyethylene oxide (PEO) and polyvinyl alcohol (PVA) is a more attractive approach than developing novel complex polymers or modifying current commercial membranes [11,15,16,17,18,19,20,21,22].

PVA is a nontoxic, biodegradable, and inexpensive polymer that is known for its excellent chemical stability, hydrophilicity, adhesive, and film-forming properties [23,24,25]. Therefore, polyvinyl alcohol is widely used in medical, commercial, and industrial applications. However, the rigid and semicrystalline structure of polyvinyl alcohol reduces its proton conductivity and subsequently its usage as a proton exchange membrane in fuel cells. Therefore, inserting doping agents or blending with another polymer electrolyte to fix this defect is important [11,16,23]. Blending of PVA with PEO is favored due to hydrogen bond formation between the –OH groups of PVA and the ether linkage of polyethylene oxide [24,26], whereas PEO is an eco-friendly polymer used in the synthesis of polymer electrolyte systems in different energy devices due to its ionic conductivity improvement, low toxicity, and flexibility [27,28].

To enhance the membrane properties many researchers have followed a common strategy of inserting doping agents into polymer structures to produce nanocomposite membranes [10,13,29,30,31]. phosphated titania (PO_4_TiO_2_) incorporation into polymer matrices is attractive in fuel cell applications as a result of its large surface area, mechanical strength, chemical stability, fuel crossover barrier, low cost, and low toxicity [23,24]. In addition to this, PO_4_TiO_2_ contains hydrophilic functional groups containing oxygen which improve water adsorption and thereby create channels for proton conduction [24]. Upon insertion of PO_4_TiO_2_ nanotubes into polymer blends, hydrogen bonds form between the –OH groups of polymer chains and oxygenated groups in PO_4_TiO_2_, these hydrogen bonds compact the membrane matrix and reinforce it, preventing excess swelling and water uptake [24,28] and are expected to enhance the membranes’ oxidative stability, sodium ion conductivity, mechanical resistance, and obstruct BH4− crossover. Further increasing the ionic conductivity of composite membranes by adding PO_4_TiO_2_ is possible, as a result of the presence of phosphate groups in their structure, which in turn increase the number of proton conducting sites.

The aim of this work was to produce novel nanocomposite membranes prepared by simple processing of biodegradable and low-cost polymers using water as a main solvent, taking a step towards DBFC commercialization. Poly vinyl alcohol was chosen as the essential polymer in the membranes due to its excellent ability to form films with PEO polymer. Then, the polymers were completely crosslinked and converted to sulfonated PVA simultaneously by using 4-sulfophthalic acid (SPA) and glutaraldehyde (GA) as crosslinkers. PO_4_TiO_2_ nanotube was synthesized and embedded as a doping agent into the polymer matrix at different concentrations to create novel nanocomposite membranes, named SPVA/PEO/PO_4_TiO_2_.

## 2. Materials and Methods

PEO (MW: 900,000 g mol-1, Acros Organics, (Fair Lawn, NJ, USA)) and PVA (99% hydrolysis and medium MW, USA). Glutaraldehyde (GA) (Alfa Aesar (Haverhill, MA, USA), 25 wt% in H_2_O) and 4-sulfophthalic acid (SPA) (Sigma-Aldrich (St. Louis, MO, USA), 99.9 wt% in H_2_O) were used as covalent and ionic cross-linkers respectively. Titanium (IV) oxide rutile (TiO2, <5 µm, ≥99.9%, Sigma-Aldrich) and H_3_PO_4_ (Fisher Chemical (Pittsburgh, PA, USA), 85 wt%).

### 2.1. Synthesis

#### 2.1.1. Synthesis of Phosphated Titanium Oxide Nanotube (PO_4_TiO_2_)

TiO_2_ nanotubes were synthesized as mentioned in a previous work [23]. TiO_2_ nanotubes were mixed to 0.1 mol/L^−^^1^ phosphoric acid in a molar ratio 1:1 and the suspension was shaken in hot water (80 °C). The mixture was washed with H_2_O and dried at 110 °C overnight. Then, the powder was burned at 450 °C in a muffle furnace.

#### 2.1.2. Synthesis of SPVA/PEO/PO_4_TiO_2_ Membranes

First, PVA (10 g) was dissolved in 100 mL deionized H_2_O at 90 °C for 2 h and PEO (2 g) was dissolved in 100 mL deionized H_2_O: Ethanol (80:20) vol% at 50 °C for 1 h then blended with PVA: PEO (85:15) wt%. After that, the crosslinked polymer was blended with GA (0.5 g, 50 wt%) as the covalent crosslinker and SPA (5 g, 99.9 wt%) as the ionic crosslinker and sulfonating agent for PVA, to convert the sample to sulfonated polyvinyl alcohol (SPVA) [20,24]. Then the inorganic–organic nanocomposite was prepared by incorporating different concentrations of PO_4_TiO_2_ nanotubes (1, 2, 3 wt% respect to PVA) in the polymeric blend as illustrated in Table 1 and named PVA/PEO, PVA/PEO/PO_4_TiO_2_-1, PVA PEO/PO_4_TiO_2_-2, PVA/PEO/PO_4_TiO_2_-3 respectively.

**Figure 1:**
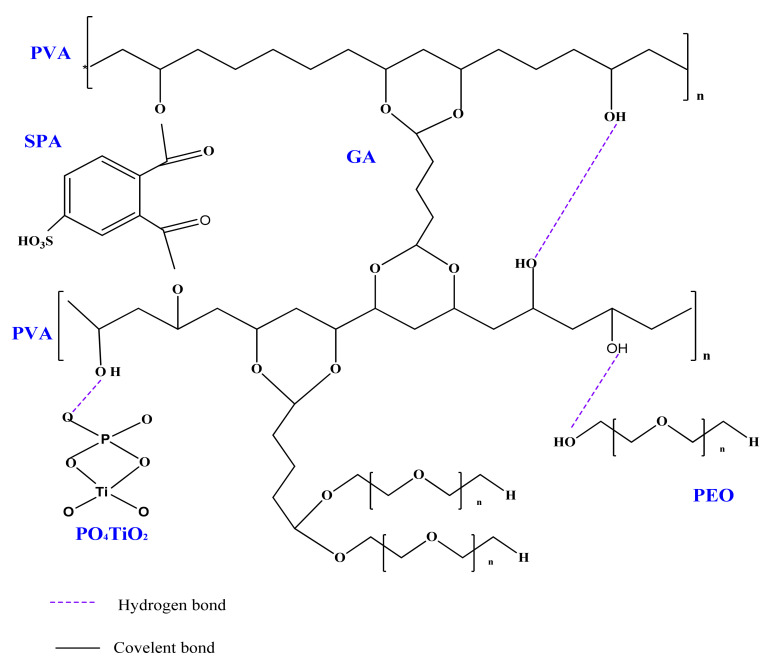
Possible structure of the SPVA/PEO/PO_4_TiO_2_ membrane.

Figure 1 shows the possible structure of SPVA/PEO/PO_4_TiO_2_ membrane, where PVA was ionically crosslinked by esterification reactions between carboxylic groups of SPA and hydroxyl groups of the polymers. In addition, the two polymers were covalently crosslinked by acetal reactions between the aldehyde groups of GA and hydroxyl groups of the polymers. Furthermore, interactions of hydrogen bonds formed between the oxygenated groups of the PO_4_TiO_2_, the –OH groups, and ether linkage of the polymers.

### 2.2. Characterization

The characteristic functional groups of PO_4_TiO_2_ nanotubes and the composite membranes were monitored by Fourier transform infrared spectrophotometer (Schimadzu FTIR-8400 S-, Kyoto, Japan), while the structures were evaluated by X-ray diffraction (Schimadzu7000-Japan). Thermal changes in the SPVA/PEO/PO_4_TiO_2_ membranes were traced using a thermogravimetric analyzer (Shimadzu TGA-50, Kyoto, Japan). The temperature range was 25–800 °C, under a nitrogen atmosphere, and the heating rate was 10 °C min^−1^. Additionally, differential scanning calorimetry (DSC) (Shimadzu DSC-60, Japan) in the range of 25–300 °C was used to evaluate the membranes. The morphological structure of the SPVA/PEO/PO_4_TiO_2_-1 membrane was shown by scanning electron microscopy (SEM). PO_4_TiO_2_ nanotube was visualized by using transmission electron microscopy (TEM, JEM 2100 electron microscope) combined with energy-dispersive X-ray analysis (EDX) (Joel Jsm 6360 LA-, Tokyo, Japan).

Contact angles between membrane surfaces and water drops were measured to evaluate the hydrophilicity of the membranes. Therefore, a contact-angle analyzer (Rame-Hart Instrument Co. model 500-FI) was used. To measure the swelling ratio (SR) and water uptake (WU), the dry membrane was cut, and its dimensions were measured and weighed. Then, the samples were placed in deionized H_2_O for one day, then dried with tissue paper and weighed again. The SR and WU of the composite membranes were calculated according to Equations (1) and (2), respectively,

(1) $$\mathrm{SR}\left(\%\right)=\frac{L_{\mathrm{wet}}{\;-\;L}_{\mathrm{dry}}}{L_{\mathrm{dry}}}\times 100$$

(2) $$\mathrm{WU}\left(\%\right)=\frac{W_{\mathrm{wet}\;}{-\;W}_{\mathrm{dry}}}{W_{\mathrm{dry}}}\times 100$$

where *L*_dry_ and *L*_wet_ are the lengths of dry and wet composite membranes, respectively, while *W*_dry_ and *W*_wet_ are the weights of dry and wet composite membranes, respectively.

The ion exchange capacity (IEC) of the prepared nanocomposite membranes was determined using acid-base titration [32]. The weighed samples were placed in 50 cm^3^ of a 2 M NaCl solution for two days, and then the solutions were titrated with a 0.01 N NaOH solution. IEC was calculated as follows:

(3) $$\mathrm{IEC}(\mathrm{meq}/g)=\frac{V_{\mathrm{NaOH}\;}{-\;C}_{\mathrm{NaOH}}}{W_{d}}\times 100$$

where *V*_NaOH_, *C*_NaOH_, and *W*_d_ are the volume of sodium hydroxide consumed in titration, the concentration of sodium hydroxide solution, and the weight of the dry sample, respectively.

To evaluate the ionic conductivity of the nanocomposite membranes, resistance measurements were evaluated by electrochemical impedance spectroscopy (EIS) using a PAR 273 A potentiostat (Princeton Applied Research, Inc. (Oak Ridge, TN, USA)) coupled to a SI 1255 HF frequency response analyzer (FRA, Schlumberger Solartron, (Leicester, UK)). First, samples were placed in 4 M NaOH solution at room temperature for half an hour, such that the test conditions were similar to those of the fuel compartment of the DBFC [23]. The membranes were placed between two stainless steel electrodes at an open circuit potential of 5 mV with signal amplitude in the 100 Hz–100 kHz frequency range. The high frequency intercept on the Nyquist plot real axis shows the bulk membrane resistance, whereas the membrane ionic conductivity was measured from the estimated resistance according to Equation (4),

(4) $$\sigma =\frac{d}{RA}$$

where σ (S cm^−1^) is the ionic conductivity of the membrane, *R* (Ω) is the membrane resistance, *A* (cm^2^) is the membrane area, and *d* (cm) is the membrane thickness.

To evaluate the borohydride permeability of the nanocomposite membrane, two small tanks of 100 mL each were placed vertically in a glass diffusion cell. The first tank, donor tank (A), was filled with 1 M NaBH_4_ in 4 M NaOH solution which is the typical DBFC anolyte, and the second tank, receptor tank (B), was filled with water [28]. Borohydride diffuses from A to B via the composite membrane as a result of the concentration difference between the two tanks, and the boron concentrations from the BH4− ions transferred to tank (B) were detected by using inductively coupled plasma–atomic emission spectrophotometer (ICP-AES, model Prodigy, Teledyne Leeman Labs (USA)). The crossover of borohydride from A to B as a function of time was determined by Equation (5),

(5) $$C_{B}(t)=\frac{A}{V_{B}}\frac{P}{L}C_{A}{(t-t}_{0})$$

where *A* (cm^2^) is the diffusion area, *V_B_* (cm^3^) is the receptor tank volume, L (cm) is the membrane thickness, *C_B_* and *C*A (mol L^−^^1^) are the borohydride concentrations in the tanks B and A, respectively, the interval (*t* − *t*_0_) is the time of the BH4− crossover and *P* is the BH4− permeability of the membrane (cm^2^ s^−^^1^). The membrane selectivity (the ratio of the ionic conductivity to the borohydride permeability) was calculated as it can provide an important indication of fuel cell performance.

The oxidative stability of fabricated membranes was measured by calculating the weight loss of the nanocomposite membrane (1.5 × 1.5 cm^2^) in Fenton’s reagent (3 wt% H_2_O_2_ containing 2 ppm FeSO_4_) at 68 °C for 24 h [23]. 

A tensile strength test, until membrane breaking, was measured for the dry nanocomposite membranes at room temperature by using Lloyd Instruments LR10k [32]. A lab fuel cell was assembled to evaluate the DBFC performance using the fabricated membranes before the performance test, and the composite membranes were placed in 0.5 M NaCl solution for one day, and then preactivated in 2 M NaOH for 4 h [28]. A platinum foil (1 cm^2^ active surface area, Metrohm) was placed in 100 mL of 1 M NaBH4 in 4 M NaOH anolyte solution. On the cathodic side, a platinum coil (~5 cm^2^) was placed in 100 mL of 5 M H_2_O_2_ in 1.5 M HCl solution [24]. The two fuel cell compartments were vertically separated by the membrane with an active area of 50 cm^2^. The fuel cell experiment was run in potentiostatic mode at room conditions. Nafion 117 was used as a commercial reference membrane for comparison purposes.

## 3. Results and Discussion

### 3.1. Characterization of PO_4_TiO_2_ Nanotube and Nanocomposite Membranes

**Figure 2:**
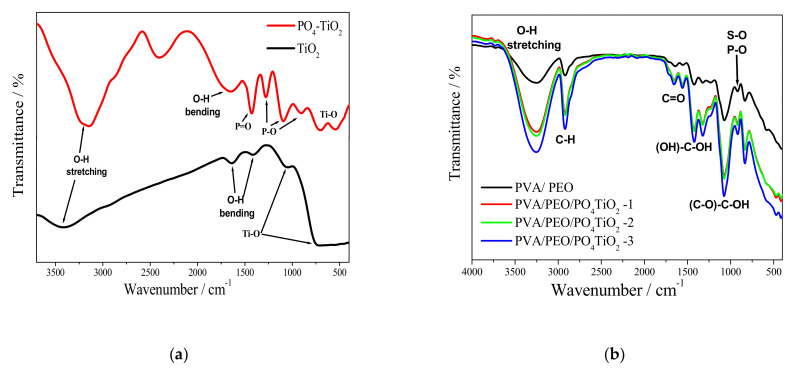
FTIR spectra of (**a**) PO_4_TiO_2_ and (**b**) PVA/PEO/PO_4_TiO_2_ membranes.

The FT-IR spectra of TiO_2_ and PO_4_TiO_2_ are shown in Figure 2a. For TiO_2_ the bands at approximately 715 and 1025 cm^−1^ are related to Ti–O bonds, and the bands at 1396, 1622 and 3387 cm^−1^ attributed to O–H bonds due to moisture adsorption on the surface of the material [33]. For PO_4_TiO_2_, the band at approximately 690 cm^−1^ corresponds to the stretching of the Ti–O bond. The bands at 890, 1085, and 1270 cm^−1^ may be attributed to the P–O bonds. The band located at approximately1425 cm^−1^ is attributed to stretching vibration of the P=O bond. The O–H bonds from H_2_O molecules adsorption are proven by the bands at approximately1630 and 3117 cm^−1^. The band located at approximately 2374 cm^−1^ may be attributed the presence of CO_2_ [34,35].

However, the FT-IR spectra of the membranes as illustrated in Figure 2b shows that the bands at approximately 3250 are characteristic bands of –OH groups of PVA and PEO while, the band at approximately 1650 cm^−1^ is attributed to the O–H bonds from water molecules that are more adsorbed as the concentration of phosphated titanium oxide increases due to its hydrophilic feature. Furthermore, the band at approximately 1112 cm^−1^ is the characteristic band of PEO [36]. However, the band at approximately 2840 cm^−1^ can be assigned to the C–H bonds in the polymers structure [37]. The characteristic peak for sulfate groups of sulfophthalic acid (SPA) is at approximately 900 cm^−1^ while the weak band at approximately 1700 cm^−1^ may be attributed to C=O bonds of the sulfophthalic acid (SPA), which proved that the crosslinking process was achieved. The band at approximately 1100 cm^−1^ may be attributed to P–O bonds of phosphated titanium oxide while the bands at approximately 1400 cm^−1^ and 1350 cm^−1^ may be attributed to the CH_3_ symmetrical deformation mode.

**Figure 3:**
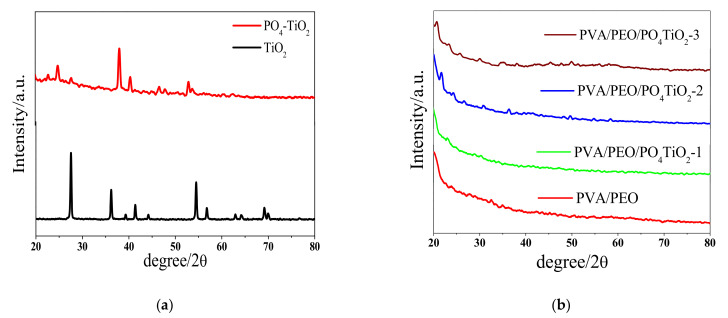
XRD patterns of (**a**) PO_4_TiO_2_and (**b**) PVA/PEO/ PO_4_TiO_2_ membranes.

Figure 3a shows the titanium oxide characteristic peaks at 2θ angle 28, 36, 41, 54 [38], however, the entry of phosphate into the titanium oxide lattice changes its original crystalline phase, whereas the sharp peaks of the original TiO_2_ at 2θ 28,° and 54° disappeared in the X-ray diffraction of PO_4_TiO_2_. Figure 3b shows the amorphous structure for the fabricated membranes which is an indication of the membrane’s ability for good conduction of ions [37].

**Figure 4:**
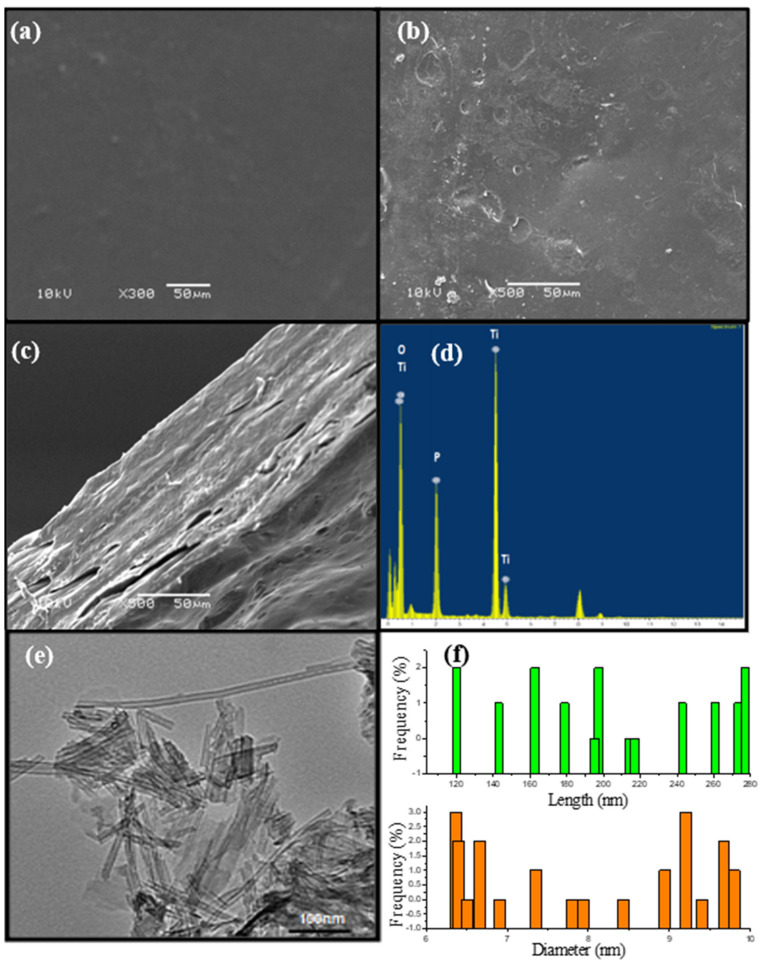
SEM images for (**a**) undoped membrane, (**b**,**c**) doped membrane (PVA/PEO/ PO_4_TiO_2_-1) surface and cross-section (**d**) EDX analysis for PO_4_TiO_2_, (**e**) TEM image for PO_4_TiO_2_ nanotubes, and (**f**) the frequency distribution plot of PO_4_TiO_2_ nanotubes size from TEM image.

The SEM images in Figure 4a,b show the surface without any defects for undoped crosslinked membrane while phosphated titanium oxide tubes clearly appear in the doped membrane, further confirmed by the EDX spectra as shown in Figure 4e. However, the SEM image in Figure 4c shows the porous structure of the cross section of the doped membrane, and consequently these voids lead to an increase in the ionic conductivity of the membranes [39]. The TEM image of phosphated titanium oxide as shown in Figure 4e proved the forming of the nanotubes shape with nanoscale size as illustrated in Figure 4f.

### 3.2. Mechanical and Thermal Analysis

The addition of TiO_2_ or functionalize TiO_2_ improves the mechanical properties of the polymeric matrix [23,24,25]. As shown in Table 2, by increasing PO_4_TiO_2_ incorporation into the polymeric matrix, the tensile strengths of the composite membranes were increased due to increasing the compatibility of the composite membrane as a result of the increase in the interaction between the two polymer functional groups, namely ether linkages, hydroxyl groups, and the characteristic phosphate groups of PO_4_TiO_2_, via hydrogen, covalent, and ionic bonds which enhanced the interfacial adhesion in the composite membranes when compared to the undoped membrane. It could be concluded that the addition of PO_4_TiO_2_ improves the mechanical tensile strength of the polymeric matrix significantly more than that of Nafion117.

**Figure 5:**
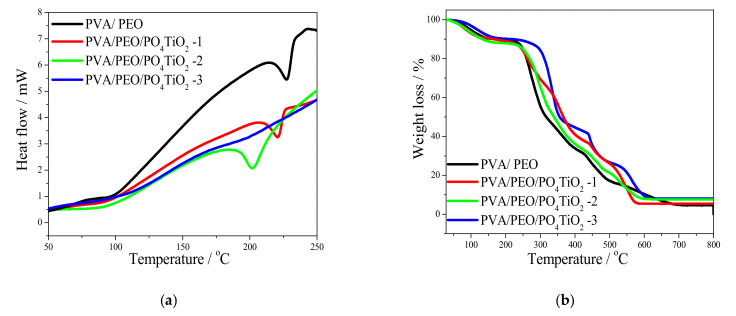
(**a**) TGA and (**b**) DSC curves of nanocomposite membranes.

The TGA curves of polymeric blend membranes without and with PO_4_TiO_2_ are illustrated in Figure 5a. The initial weight loss at ~150 °C (~10%) that may be attributed to moisture evaporation in all membranes [40]. The second weight loss of composite membranes occurred in the range of ~150–300 °C and may be attributed to functional group degradation [41,42]. The third weight loss stage appeared as a marked decomposition from ~300–580 °C and may be referred to as polymeric chain decomposition [43], which started at 250 °C for the undoped membrane, while for the doped membranes, with 3 wt% doping, it started at 310 °C with lower weight percentage. This behavior clarifies that PO_4_TiO_2_ incorporation enhances the thermal stability of composite membranes by increasing the hydrogen bonding in the composite. For the DSC curves, as shown in Figure 5b, the existence of only one endothermic peak provides a proof of complete miscibility in the membrane structure, and the disappearance of this peak at PO_4_TiO_2_ (3 wt%) may be attributed to the formation of many hydrogen bonds between the doping agent and polymer structure [29]. The melting temperature of the membranes decreased with increasing doping agent concentration. This behavior could be explained by the hydrogen bond interactions which partially destroy the membrane crystallinity, that in turn reduces the melting point and enhances the ionic conductivity [29].

The behavior of the composite membranes in contact with deionized water is shown in Table 2. The membrane surfaces are considered hydrophobic when the contact angle is ≥90° and hydrophilic when the contact angle is <90°. The composite membranes have a less hydrophilic quality with more thickness because of the increased doping agent content [28,43]. It was also noticed that, as the amount of PO_4_TiO_2_ increased in the polymeric matrix from 1% to 3% the swelling ratio and water uptake of the polymeric membranes decreased, which is very necessary as water overload can be avoided [44]. In other words, increasing the doping agent in the membrane matrix leads to an increase in the compactness of the structure, which in turn avoids water overload in the polymeric matrix channels when compared with undoped membranes [45,46].

### 3.3. Oxidative Stability

The chemical stability of the nanocomposite membranes as illustrated in Table 2, was measured by a Fenton’s reagent test. An undoped membrane gives the lowest chemical stability, while introducing PO_4_TiO_2_ as a dopant enhances the membrane resistance to OOH and OH radical attack. PVA/PEO/PO_4_TiO_2_-3 membrane was the most stable fabricated membrane at which its retained weight was approximately 99% and which gives proof of the addition of the doping agent such as TiO_2_ or functionalized TiO_2_ in increasing the chemical stability of the polymeric membranes [24,47].

### 3.4. Ionic Conductivity, IEC, and Borohydride Crossover

**Figure 6:**
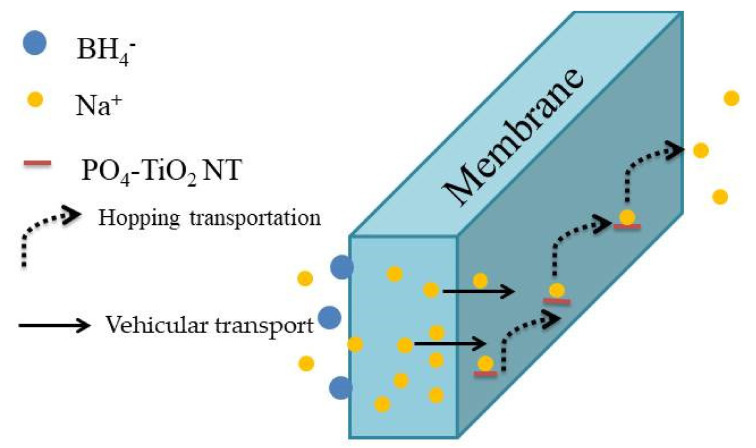
Schematic illustration for the ion transportation mechanism.

The IEC values are presented in Table 3, and it can be observed that as the amount of PO_4_TiO_2_ increases in the composite membranes, the IEC values increase, because the polymeric matrix contains more acidic exchangeable groups from PO_4_TiO_2_. This is directly related to the good ionic conductivity of SPVA/PEO/PO_4_TiO_2_-3 (28 mS cm^−1^) when compared with the undoped membrane (12 mS cm^−1^); the acidic (phosphate) sites of PO_4_TiO_2_ increase the charges in the polymeric matrix, which in turn enhance its ionic conduction with Na+ transfer by hopping on the acidic sites of PO_4_TiO_2_ “Grotthuss mechanism” while also Na+ ions transfer across the membranes using vehicular transport via hydrogen bonds formed between polymers function groups and PO_4_TiO_2_ [23,24] as shown in Figure 6. Regarding the fuel permeability of composite membranes, it can be seen that the introduction of PO_4_TiO_2_ into the polymeric matrix obstructs BH4− crossover. As illustrated in Table 3, the BH4− permeability of the undoped polymeric membrane was 16 × 10^−6^ cm^2^ s^−1^ and upon incorporation of PO_4_TiO_2_ into the membrane matrix, the permeability decreased to a value of 0.10 × 10^−6^ cm^2^ s^−1^ for the SPVA/PEO/PO_4_TiO_2_-3, while Nafion 117 achieved a much higher value (0.40 × 10^−6^ cm^2^ s^−1^). The decrease in the BH4− permeability of the membrane containing the doping agent may be attributed to the ability of the doping agent to narrow the polymeric matrix channels that decrease the water uptake, and thus, the fuel permeability is reduced [23,24,48,49]. The higher selectivity was noted for SPVA/PEO/PO_4_TiO_2_-3 which was 2.8 × 10^5^ S cm^−3^ s compared to undoped SPVA/PEO membrane which has selectivity approximately 0.007 × 10^5^ S cm^−3^ s or Nafion 117 which was 1.12 × 10^5^ S cm^−3^ s. This is an indication of the suitability of the fabricated nanocomposite membranes to be used in DBFCs [45].

### 3.5. Fuel Cell Performance

**Figure 7:**
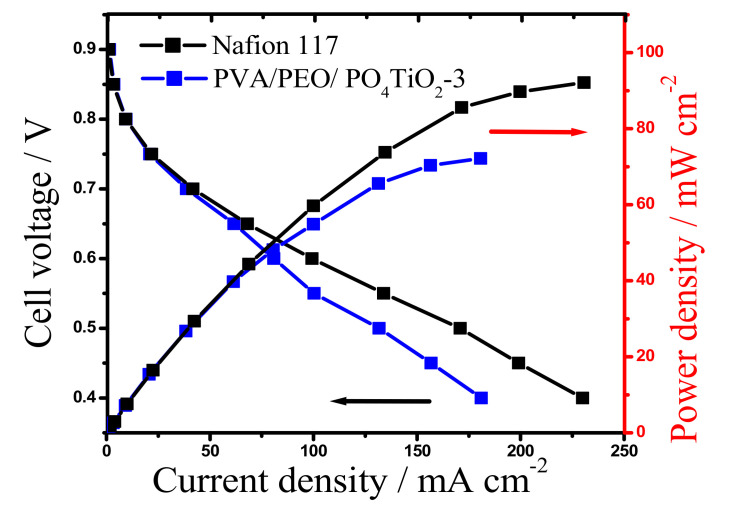
Power density curves and polarization of DBFCs using PVA/PEO/PO_4_TiO_2_-3 and Nafion117 membranes, at room temperature.

The best nanocomposite membrane which realized physicochemical properties better than Nafion117 was tested in a lab DBFC and compared with the performance of Nafion117 membrane with the same dimensions under the same test conditions. The polarization curves as illustrated in Figure 7 show that, PVA/PEO/PO_4_TiO_2_-3 membrane leads to lower DBFC discharge currents than Nafion117, which may be because the charge density of Nafion117 is higher and, to some extent the electrochemical reactions at the cathode and anode are limited by Na^+^ ions mass transfer through the PVA/PEO/PO_4_TiO_2_-3 membrane [24,28]. However, the resulting peak power density for DBFC with PVA/PEO/PO_4_TiO_2_-3 (72 mW cm^−2^) is very near to the value of DBFC with Nafion117 (91 mW cm^−2^).

## 4. Conclusions

A less expensive nanocomposite membrane was successfully prepared through a simple blending and solution casting method using eco-environmental and available polymers. The incorporation of PO_4_TiO_2_ nanotubes as doping agent into the polymer blend improves the membrane properties, namely, ionic conductivity, mechanical properties, oxidative stability, reduction of water overload, while the BH4− crossover limiting was enhanced, especially in the composite membrane with 3 wt% of PO_4_TiO_2._ This showed oxidative stability and tensile strength better than Nafion117 and fuel permeability less than Nafion 117 besides achieving water uptake and swelling ratio nearly equal to Nafion117 values. The power density also with PVA/PEO/PO_4_TiO_2_-3 was 72 mW cm^−^^2^, which was close to Nafion117 (91 mW cm^−^^2^) under the same test conditions. In conclusion, the prepared membrane with optimum properties needs several further simple modifications to compete and replace the Nafion membrane in DBFCs and to be considered as an efficient progression step towards development of green and low cost DBFCs.

## Figures and Tables

**Table 1:** Membranes composition.

Membrane	PVA: PEO wt%	PO_4_-TiO_2_ Nanotubes wt% Respect to PVA
SPVA/PEO	85:15	0
SPVA/PEO/PO_4_TiO_2_-1	85:15	1
SPVA/PEO/PO_4_TiO_2_- 2	85:15	2
SPVA/PEO/PO_4_TiO_2_- 3	85:15	3

**Table 2:** Physicochemical properties of the fabricated membranes and Nafion 117 [28].

Membrane	Thickness (µm)	WU (%)	SR (%)	Contact Angle (°)	Tensile Strength (MPa)	Oxidative Stability (RW, %) *
SPVA/PEO	130	95 ± 0.5 **	90 ± 0.3	65.36 ± 1.5°	15.5 ± 0.5	90 ± 2
SPVA/PEO/PO_4_TiO_2_-1	150	40 ± 0.3	42 ± 0.3	67.23 ± 1.5°	24.9 ± 0.7	94 ± 1.5
SPVA/PEO/PO_4_TiO_2_-2	175	22 ± 0.2	13 ± 0.2	70.36 ± 1.7°	32.5 ± 1	98 ± 1.5
SPVA/PEO/PO_4_TiO_2_-3	184	16 ± 0.03	10 ± 0.1	72.30 ± 1.5°	40.3 ± 1.5	99 ± 0.5
Nafion 117	183	15	8	102	25	92

* The retained weight of membranes (RW) after immersion for a day in Fenton’s reagent. ** The measurements were replicated three times for the same prepared membranes and the standard deviation was evaluated accordingly for all tests.

**Table 3:** Ionic conductivity, borohydride permeability, IEC, and selectivity of the fabricated membranes and Nafion 117 [28].

Membrane	IEC (meq g^−1^)	Ionic Conductivity (mS cm^−1^)	Borohydride Permeability (10^−6^ cm^2^ s^−1^)	Selectivity (10^5^ S cm^−3^ s)
SPVA/PEO	0.20 ± 0.01 *	12 ± 0.05	16	0.007
SPVA/PEO/PO_4_TiO_2_-1	0.35 ± 0.01	17.7 ± 0.05	0.75	0.23
SPVA/PEO/PO_4_TiO_2_-2	0.45 ± 0.01	20.5 ± 0.05	0.36	0.56
SPVA/PEO/PO_4_TiO_2_-3	0.60 ± 0.01	28 ± 0.03	0.10	2.80
Nafion 117	0.89	45.0	0.40	1.12

* The measurements were replicated three times for the same prepared membranes and the standard deviation was evaluated accordingly for all tests.
